# Mapping the Adolescent Mind: Profiles of Reflective Functioning and Epistemic Trust in Adolescent Psychopathology

**DOI:** 10.1002/jad.70160

**Published:** 2026-04-19

**Authors:** Ilaria Maria Antonietta Benzi, Nick Midgley, Karin Ensink, Laura Parolin, Peter Fonagy, Francesca Locati

**Affiliations:** ^1^ Department of Psychology University of Milan‐Bicocca Milan Italy; ^2^ Research Department of Clinical, Educational and Health Psychology University College London London UK; ^3^ Department of Psychology Laval University Quebec City Quebec Canada; ^4^ Department of Humanities University of Pavia Pavia Italy

**Keywords:** adolescence, epistemic trust, externalizing, internalizing, latent profile analysis, reflective functioning

## Abstract

**Introduction:**

Adolescence is a period of heightened vulnerability to psychopathology, when capacities for mentalising and interpersonal trust develop rapidly. This study investigated how configurations of reflective functioning (RF) and epistemic trust (ET) differentiate patterns of internalizing and externalizing problems in youth.

**Methods:**

In a community sample of 688 adolescents (47.8% female; *M* = 15.51 years, SD = 1.80), latent profile analysis was conducted on RF uncertainty and certainty, as well as ET trust, mistrust, and credulity.

**Results:**

Four distinct profiles were identified. An Ambivalent profile (intact RF with high credulity and mistrust; mainly female) was associated with the highest internalizing problems, while an Impaired RF profile (simultaneously high uncertainty and certainty; mainly male) showed the highest externalizing problems. An Adaptive profile (older adolescents with low RF impairment, high trust, and low mistrust/credulity) and a Disengaged profile (consistently low RF and ET scores) exhibited the lowest levels of symptoms. A MANOVA confirmed significant group differences across profiles.

**Conclusions:**

These findings suggest that different RF–ET configurations reflect complementary pathways to internalizing and externalizing vulnerabilities, highlighting the importance of person‐centered assessment for identifying process‐based targets for early prevention and intervention.

## 1 Introduction

Mentalisation, operationalised as reflective functioning (RF), refers to the ability to interpret one's own and others' behaviors as stemming from intentional inner mental states (Bateman and Fonagy 2016; Fonagy and Luyten 2012; Luyten et al. 2020). It is a multidimensional ability encompassing four polarities—implicit/explicit, self/other, internal/external, and cognitive/affective—that develops most effectively within secure attachment relationships (Allen 2018; Fonagy and Luyten 2009). Although its foundations are established in childhood, adolescence is a sensitive period for sociocultural processing, supported by the maturation of the social brain (Blakemore and Mills 2014). During this stage, adolescents must form a coherent identity and navigate increasingly complex peer and romantic relationships (Benzi, Fontana, et al. 2023; Ensink et al. 2024). Mentalising ability follows a curvilinear trajectory, continuing to develop through adolescence and reaching a peak in young adulthood (Desatnik et al. 2023). Strong mentalising skills act as a protective factor against psychopathology, whereas impairments serve as a transdiagnostic risk for both internalizing and externalizing difficulties (Luyten et al. 2020). However, the literature on mentalising and psychopathology covers child, adolescent, and adult samples, and findings from these developmental stages cannot be assumed to generalize directly to adolescence.

Impaired mentalising has been linked to a range of psychopathological outcomes across developmental and clinical studies, with adolescent research pointing in particular to externalizing difficulties (Luyten et al. 2020; Zavlis et al. 2025). Difficulties in RF have been consistently connected to substance use disorder (Suchman et al. 2018), pathological gambling (Ciccarelli et al. 2021), attention deficit hyperactivity disorder (Poznyak et al. 2019), and conduct and oppositional disorders (Abate et al. 2017; Adler et al. 2021). Compared to non‐clinical groups, adolescents with disruptive behavior disorders show significant struggles in understanding their own and others' mental states (Bizzi et al. 2020). Theoretical accounts propose that impaired mentalising reduces perspective‐taking and the ability to assess the consequences of one's actions on others, thereby encouraging disruptive and aggressive behaviors (Fonagy and Luyten 2009; Tironi et al. 2024). These impairments may appear in various forms; for example, hyper‐mentalising—the tendency to make distorted, overly confident assumptions about others' intentions, often following early maltreatment—is linked to increased aggression (Allen 2018; Locati, Benzi, et al. 2023; Luyten and Fonagy 2019). Importantly, evidence from adolescent studies does not rely exclusively on self‐report measures. For example, Taubner et al. (2016) found that mentalization mediated the association between early maltreatment and potential for violence in adolescence, while Protić et al. (2020) reported differences in attachment dimensions and RF between traumatized juvenile offenders and maltreated non‐delinquent adolescents in care services (Protić et al. 2020; Taubner et al. 2016).

Beyond externalizing problems, deficits in mentalising are also closely linked to internalizing symptoms, including depression, anxiety, and somatic complaints (Ensink et al. 2016; Locati, Benzi, et al. 2023; Malenka et al. 2026; Zavlis et al. 2025). Such difficulties interfere with emotion regulation, a core process in the onset and maintenance of internalizing disorders (Parolin et al. 2024). Yet the association is nuanced; some evidence suggests that certain RF styles may paradoxically exacerbate internalizing symptoms (Locati, Benzi, et al. 2023). Adolescents with stronger mentalising skills may be more vulnerable to rumination and overthinking, amplifying anxious and depressive affect. This highlights that it is not simply the presence or absence of mentalising that matters but its quality, flexibility, and adaptability, which are central to mental health.

The developmental framework of mentalisation is closely linked to epistemic Trust (ET), defined as the capacity to evaluate and accept interpersonally transmitted knowledge as accurate, reliable, and personally relevant (Fonagy and Allison 2014). ET is not a singular construct but comprises three interconnected dimensions: adaptive Trust, which facilitates social learning; maladaptive Mistrust, characterized by rigid suspicion of others' communications; and maladaptive Credulity, a naive and indiscriminate acceptance of information that increases vulnerability to exploitation (Campbell et al. 2021; Li et al. 2023). Growing evidence shows that although adaptive Trust plays a nuanced role, maladaptive Mistrust and Credulity are consistently associated with increased psychopathology and are strongly linked to the lasting effects of adverse childhood experiences.

Mentalising is described as the “means by which epistemic trust is established” (Fonagy and Allison 2014, p. 375), enabling individuals to recognize those invested in their well‐being, reduce epistemic vigilance, and engage more fully in social learning (Fonagy and Allison 2023). This is especially important during adolescence, when interest in one's own and others' minds develops, creating new opportunities for acquiring social knowledge (Blakemore and Mills 2014). Learning through relational exchanges at this stage supports the development of key capacities such as emotion regulation (Parolin et al. 2024). However, disruptions in ET, often rooted in early adversity including abuse and neglect, are consistently linked with internalizing and externalizing psychopathology (Benzi, Carone, et al. 2023, Fonagy and Allison 2023; Knapen et al. 2025; Locati, Milesi, et al. 2023; Orme et al. 2019). Recent research examining ET in clinical and distressing conditions (Campbell et al. 2021; Jaffrani et al. 2020) indicates that such disruptions can entrench negative communication patterns (Nolte et al. 2023), distort interactions, and bias appraisal mechanisms towards negativity while undermining positive responses (Li et al. 2023). For example, anxiety disorders may involve epistemic freezing, with rigid reliance on prior knowledge to minimize stress. Conversely, conduct disorder may be characterized by a lack of ET, supporting aggression as a means of communication (Talia et al. 2021).

Although RF, ET, and psychopathology are conceptually connected, relatively few studies have examined their interplay specifically during adolescence, particularly with respect to the three components of ET—Trust, Mistrust, and Credulity. More broadly, while foundational developmental models often draw on child research and emerging evidence on ET has also been informed by adult clinical samples, adolescent‐specific evidence remains limited. This is a meaningful gap, given that adolescence is a particularly sensitive developmental period for social learning, identity formation, and the reorganization of self–other understanding (Fonagy and Allison 2023; Stagaki et al. 2022; Zavlis et al. 2025).

This indicates that profiles, as specific arrangements of mentalising abilities and epistemic stances, may predict outcomes more effectively than single variables alone. A person‐centered approach, which views individuals as integrated wholes rather than collections of independent traits, is therefore essential to capture these subtle pathways. Evidence from LPA in a large adult clinical sample (Benzi et al. 2025) demonstrated the usefulness of transdiagnostic profiling based on ET and emotion regulation, emphasizing its potential to inform personalized psychological interventions.

### 1.1 The Present Study

Although the theoretical connections between RF, ET, and psychopathology are well understood, few studies have explored these processes together in adolescence, especially concerning the three components of ET—Trust, Mistrust, and Credulity. Variable‐centered approaches might also overlook how different combinations of strengths and vulnerabilities in RF and ET shape distinct developmental pathways. Identifying these naturally occurring profiles is, therefore, an essential step toward understanding psychopathological risk and guiding tailored interventions.

To address this gap, we used a person‐centered approach. Our goals were to identify distinct, data‐driven profiles of adolescents based on patterns of RF and ET; to characterize these profiles in terms of age and gender; and to examine their relationships with internalizing and externalizing psychopathology.

Drawing on previous theory and empirical research, we hypothesized that multiple profiles would emerge. We expected to find an Adaptive profile, characterized by high RF and high ET. Additionally, consistent with theory on maladaptive epistemic stances, we predicted profiles marked by high Mistrust and/or Credulity, including one combining both high Mistrust and high Credulity, a pattern observed in clinical populations (Campbell et al. 2021). Following recent studies (Martin‐Gagnon et al. 2023), we also anticipated a profile defined by high levels of both Certainty and Uncertainty about mental states, reflecting confusion about one's own mental states alongside excessive confidence in others'. We further expected that the profiles would vary in their associations with symptoms. Specifically, the Adaptive profile would show the lowest levels of both internalizing and externalizing problems. Conversely, profiles characterized by high Mistrust, Credulity, and/or Uncertainty were expected to exhibit higher symptoms.

## 2 Materials and Methods

### 2.1 Participants and Procedure

The sample comprised 688 cisgender adolescents recruited from secondary schools across Italy, including 359 males (52.2%) and 329 females (47.8%). Participants ranged in age from 12 to 19 years (*M* = 15.51, SD = 1.80). Approval for the study was obtained from school administrators, and informed consent was provided by parents, with adolescents giving their own assent. Each participant received a unique reference code to ensure anonymity, was assured of confidentiality, and was informed of their right to withdraw at any time without penalty. Data were collected via self‐report questionnaires administered through a private Qualtrics link. The study complied with the ethical standards of the American Psychological Association and the Declaration of Helsinki. The institutional review board of the University of Milano‐Bicocca approved all procedures.

### 2.2 Measures

RF was assessed with the 13‐item Reflective Functioning Questionnaire for Youth (RFQY‐13; Martin‐Gagnon et al. 2023). Items are rated on a 6‐point Likert scale from 1 (*strongly disagree*) to 6 (*strongly agree*). Two dimensions of mentalising impairment were used: Uncertainty about mental states, reflecting difficulties in attributing mental states and leading to doubt about one's own and others' thoughts and feelings (e.g., *“I don't always know why I do what I do”*); and Certainty about mental states, reflecting excessive and rigid confidence that can result in over‐interpretation of social cues (e.g., *“I usually know exactly what other people are thinking*”). Internal consistency was good to excellent, with *α* = 0.88 for Uncertainty and *α* = 0.83 for Certainty.

Epistemic stances were measured with the Epistemic Trust, Mistrust, and Credulity Questionnaire (ETMCQ; Campbell et al. 2021; Liotti et al. 2023). This 15‐item self‐report measure uses a 7‐point Likert scale from 1 (*strongly disagree*) to 7 (*strongly agree*). It yields three dimensions: Trust, reflecting adaptive openness to social learning; Mistrust, reflecting a tendency to perceive others' information as unreliable or malicious; and Credulity, reflecting a lack of discrimination that increases vulnerability to misinformation. Internal consistency was acceptable, with *α* = 0.66 for Trust, *α* = 0.67 for Mistrust, and *α* = 0.75 for Credulity.

Psychopathology was assessed with the Youth Self‐Report (YSR/11–18; Achenbach and Rescorla 2001), a 112‐item questionnaire where adolescents rate items on a 3‐point scale (0 = *not true*, 1 = *somewhat or sometimes true*, 2 = *very true or often true*). Two broadband scales were used: Internalizing Problems, reflecting anxious, depressive, and somatic symptoms, and Externalizing Problems, reflecting rule‐breaking and aggressive behaviors. Higher scores indicate greater symptom severity. Internal consistency was good to excellent, with *α* = 0.90 for Internalizing and *α* = 0.80 for Externalizing.

### 2.3 Statistical Analyses

Analyses were conducted in R (Version 4.5.1; Posit team, 2025; R Core Team, 2025) following a two‐stage person‐centered approach. To provide a variable‐centered benchmark against which to interpret the person‐centered findings, a series of mediation analyses were conducted using the psych package in R. We tested whether ET variables (Mistrust and Credulity) mediated the relationship between RF (Uncertainty and certainty) and psychopathology outcomes (internalizing and externalizing problems), using bootstrapping with 5000 iterations to generate robust confidence intervals for the indirect effects.

First, latent profile analysis (LPA) was performed using the *mclust* package (Fraley et al. 2012) to identify subgroups of adolescents based on standardized scores for the five indicators: RFQ‐Uncertainty (RFQ‐U), RFQ‐Certainty (RFQ‐C), Trust, Mistrust, and Credulity. Models with one to five profiles were estimated. Model selection drew on several criteria: Bayesian Information Criterion (BIC; lower values indicating better fit), the Bootstrap Likelihood Ratio Test (BLRT; testing whether a k‐profile model significantly improves on a k–1 model), and the theoretical coherence and interpretability of the profiles (Muthén and Muthén 2019).

Second, after selecting the optimal solution, validation analyses were conducted. Chi‐square tests assessed gender distribution across profiles, and one‐way ANOVA tested for mean age differences. Differences in psychopathology outcomes were examined with one‐way MANOVA, using profile membership as the independent variable and YSR Internalizing and Externalizing scores as dependent variables. A significant omnibus MANOVA was followed by univariate ANOVAs and Tukey's HSD post hoc tests. The alpha level was set at 0.05 for all analyses.

## 3 Results

Descriptive statistics for the main study variables are reported in Table 1, with bivariate correlations shown in Table 2. RF and ET variables were largely unrelated. RFQ‐U and RFQ‐C were strongly and positively correlated. Mistrust and Credulity showed a moderate positive association, and Trust correlated weakly but positively with Credulity. In relation to psychopathology, higher levels of both Mistrust and Credulity were moderately to strongly associated with higher Internalizing scores. Mistrust was also moderately associated with higher Externalizing scores. Both RFQ‐U and RFQ‐C showed small but significant positive correlations with Externalizing problems.

**Table 1:** Descriptive statistics for the main study variables.

Variable	*M*	SD
Age	15.51	1.80
RFQ Uncertainty (RFQ‐U)	2.74	1.14
RFQ Certainty (RFQ‐C)	2.83	1.01
ETMCQ Trust	4.71	1.05
ETMCQ Mistrust	4.50	1.05
ETMCQ Credulity	3.38	1.31
YSR Internalizing	20.38	11.37
YSR Externalizing	17.35	7.35

*Note:* M = mean, SD = standard deviation.

**Table 2:** Bivariate correlations among the main study variables.

Variable	1	2	3	4	5	6	7	8
1. Age								
2. RFQ‐U	−0.02							
3. RFQ‐C	−0.01	0.83**						
4. Trust	0.15**	−0.02	0.03					
5. Mistrust	0.05	0.00	0.04	0.11**				
6. Credulity	−0.03	0.00	0.08*	0.22**	0.34**			
7. YSR‐Internalizing	0.01	0.08*	0.12**	0.01	0.50**	0.36**		
8. YSR‐Externalizing	−0.01	0.26**	0.25**	−0.04	0.27**	0.16**	0.31**	

*Note:* *p < 0.05, **p < 0.01.

Abbreviations: RFQ‐C, Reflective Functioning Certainty; RFQ‐U, Reflective Functioning Uncertainty; YSR, Youth Self‐Report.

### 3.1 Variable‐Centered Benchmark Analysis

To establish a variable‐centered benchmark for the primary person‐centered analyses, we tested a series of mediation models. None of the models showed a significant indirect effect (denoted as ab), as all bootstrapped confidence intervals included zero. The pathway from RF Uncertainty to Internalizing problems was not mediated by Mistrust (ab = 0.01, 95% CI = [−0.37, 0.39]) or Credulity (ab = 0.00, 95% CI = [−0.29, 0.28]). Similarly, the pathway to Externalizing problems was not significantly mediated by Mistrust (from RF Certainty; ab = 0.07, 95% CI = [−0.08, 0.21]) or Credulity (from RF Uncertainty; ab = 0.00, 95% CI = [−0.09, 0.08]). The absence of significant indirect effects suggests that these relationships are not adequately captured by simple linear mediation models, supporting the use of a more nuanced, person‐centered approach.

### 3.2 Latent Profile Analysis

LPA was conducted to identify subgroups of adolescents based on RFQ‐U, RFQ‐C, Trust, Mistrust, and Credulity. Competing profile solutions were compared using BIC, BLRT, entropy, and theoretical interpretability. As shown in Table 3, the 5‐profile solution did not significantly improve on the 4‐profile model (*p* = 0.984). The 4‐profile model also showed acceptable classification quality (entropy = 0.77) and was theoretically interpretable. On both statistical and theoretical grounds, the 4‐profile solution was retained.

**Table 3:** Model fit statistics for latent profile analysis.

N Profiles	BIC	ICL	Entropy	BLRT *p*‐value
2	−8437.94	−8599.99	0.74	< 0.001
3	−8565.50	−8789.26	0.77	< 0.001
**4**	−**8576.70**	−**8818.11**	**0.77**	**< 0.001**
5	−8593.99	−8887.31	0.77	0.984

*Note:* The best‐fitting model is in bold. BLRT *p*‐value compares the fit of the k‐profile model to the k‐1 profile model.

Abbreviations: BIC, Bayesian Information Criterion; ICL, Integrated Completed Likelihood.

**Figure 1:**
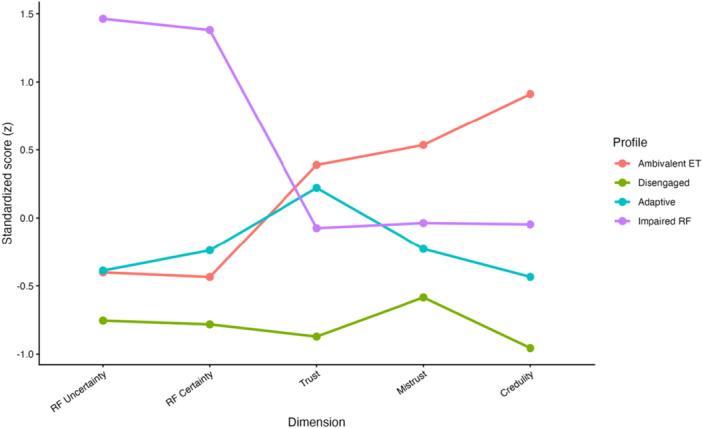
Patterns of reflective functioning and epistemic trust across profiles. *Note:* The figure displays standardized (z) scores for the five indicator variables across the four latent profiles. Profile labels correspond to the following numerical classifications used in the analyses: Profile 1 = Ambivalent ET; Profile 2 = Disengaged; Profile 3 = Adaptive; Profile 4 = Impaired RF.

Profiles were defined by distinct standardized mean scores on the five indicators (see Figure 1; raw means in Table 4). Profile 1 (Ambivalent epistemic stances; *n* = 222, 32.3%) was marked by below‐average RFQ‐U (−0.40 SD) and RFQ‐C (−0.44 SD), with elevated Credulity (+0.91 SD), Mistrust (+0.54 SD), and Trust (+0.39 SD). Profile 2 (Disengaged; *n* = 127, 18.5%) showed the lowest scores across all variables: RFQ‐U (−0.75 SD), RFQ‐C (−0.78 SD), Trust (−0.87 SD), Mistrust (−0.58 SD), and Credulity (−0.96 SD). Profile 3 (Adaptive; *n* = 168, 24.4%) showed low RFQ‐U (−0.39 SD) and RFQ‐C (−0.24 SD), slightly above‐average Trust (+0.22 SD), the lowest Mistrust (−0.23 SD), and below‐average Credulity (−0.44 SD). Profile 4 (Impaired RF; *n* = 171, 24.9%) was defined by markedly above‐average RFQ‐U (+1.46 SD) and RFQ‐C (+1.38 SD), with Trust (−0.07 SD), Mistrust (−0.04 SD), and Credulity (−0.05 SD) close to the sample mean.

**Table 4:** Unstandardized means of LPA indicators by profile.

Profile	RFQ‐U	RFQ‐C	Trust	Mistrust	Credulity
Profile 1 (Ambivalent ET)	2.28	2.39	5.12	5.06	4.58
Profile 2 (Disengaged)	1.88	2.04	3.79	3.89	2.13
Profile 3 (Adaptive)	2.30	2.59	4.95	4.26	2.81
Profile 4 (Impaired RF)	4.40	4.23	4.63	4.46	3.32

Abbreviations: RFQ‐C, Reflective Functioning Certainty; RFQ‐U, Reflective Functioning Uncertainty.

### 3.3 Profile Validation and Associations With Psychopathology

Chi‐square tests showed significant gender differences across profiles, *χ* ^2^(3, *N* = 688) = 82.88, *p* < 0.001. Females were overrepresented in Profile 1 (Ambivalent epistemic stances; 69.8%) and underrepresented in Profile 4 (Impaired RF; 26.3%). Males were overrepresented in Profile 4 (73.7%) and underrepresented in Profile 1 (30.2%). Profile 2 (Disengaged) showed a male predominance (64.6%), while Profile 3 (Adaptive) had equal gender distribution (50% each). ASR values confirmed that female overrepresentation in Profile 1 (ASR = +4.74) and male overrepresentation in Profile 4 (ASR = +3.89) exceeded the ±3.0 threshold, indicating meaningful deviations from expected frequencies. Profile 2 showed a smaller male predominance (ASR = +1.93), below conventional significance levels.

A one‐way ANOVA revealed significant age differences across the profiles, *F*_(3, 684)_ = 6.06, *p* < 0.001. Mean ages were Profile 1, *M* = 15.64, SD = 2.02; Profile 2, *M* = 15.10, SD = 1.89; Profile 3, *M* = 15.88, SD = 2.00; and Profile 4, *M* = 15.27, SD = 0.94. Post hoc Tukey tests indicated that Profile 3 (Adaptive) participants were significantly older than Profile 2 (*p* = 0.001), and Profile 2 participants were significantly younger than Profile 1 (*p* = 0.037). Profile 4 participants were also significantly younger than Profile 3 (*p* = 0.009). No other pairwise comparisons reached significance.

**Figure 2:**
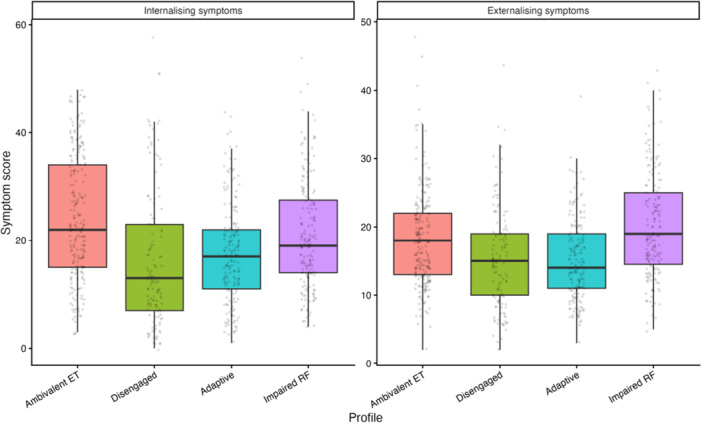
Differences in psychological symptoms across profiles. *Note:* The boxplots show the distribution of raw scores for YSR Externalizing and Internalizing problems across the four latent profiles. The horizontal line represents the median, the box represents the interquartile range (IQR), and the whiskers extend to 1.5 times the IQR. Profile labels correspond to the numerical classifications used in the analyses: Profile 1 = Ambivalent ET; Profile 2 = Disengaged; Profile 3 = Adaptive; Profile 4 = Impaired RF.

To test the primary hypothesis, a one‐way MANOVA was conducted on psychopathology outcomes (YSR Internalizing and Externalizing scores) across the four profiles (see Figure 2). The omnibus test was significant, Pillai's Trace = 0.13, *F*_(6, 1368)_ = 16.16, *p* < 0.001, indicating overall group differences.

Follow‐up univariate ANOVAs were significant for both Internalizing, *F*_(3, 684)_ = 18.71, *p* < 0.001, η² = 0.08, and Externalizing problems, *F*_(3, 684)_ = 18.97, *p* < 0.001, η² = 0.08. Tukey's HSD post hoc comparisons showed that for Internalizing problems, Profile 1 reported the highest scores, significantly greater than all other profiles (*p*s < 0.02). Profiles 2 and 3 had the lowest scores and did not differ. Profile 4 fell in between, scoring significantly higher than Profiles 2 (*p* = 0.002) and 3 (*p* = 0.022).

For Externalizing problems, Profile 4 reported the highest scores, significantly greater than all other profiles (*p*s < 0.03). Profiles 2 and 3 again had the lowest scores and did not differ. Profile 1 scored in the mid‐range, significantly higher than Profiles 2 (*p* = 0.002) and 3 (*p* < 0.001), but lower than Profile 4.

## 4 Discussion

This study used a person‐centered approach to explore the relationship between RF and ET in a large community sample of adolescents, aiming to identify distinct social–cognitive profiles and examine their associations with psychopathology. Consistent with our hypotheses, four unique profiles were identified, each showing different associations with internalizing and externalizing problems. The results revealed greater complexity than expected, providing new insights into developmental pathways of risk during adolescence (Blakemore and Mills 2014; Benzi, Carone, et al. 2023; Benzi et al. 2025). The four‐profile solution offers strong empirical support for the idea that adolescents adopt distinct, coherent strategies for navigating their social worlds, with profiles reflecting unique combinations of mentalising capacities and epistemic stances.

Before detailing each profile, it is noteworthy to highlight the demographic and developmental patterns that emerged. The profiles were not evenly distributed by gender, with females being significantly overrepresented in the Ambivalent profile and males in the Impaired RF profile. The Adaptive profile showed a balanced gender distribution. Age also distinguished the profiles: participants in the Adaptive profile were, on average, the oldest, while those in the Disengaged and Impaired RF groups were considerably younger, suggesting some profiles may reflect less mature social‐cognitive styles.

The Ambivalent epistemic stances profile (Profile 1), the largest group (32.3%), exhibited a paradoxical configuration. These adolescents reported relatively intact RF but simultaneously endorsed high levels of both Trust and Mistrust, with Credulity emerging as the most prominent feature. Credulity signifies an excessive or uncritical form of trust (Campbell et al. 2021), reflecting openness to information without adequate evaluation. When combined with high Mistrust, this pattern may indicate a state of epistemic ambivalence, where a tendency for uncritical acceptance of some information coexists with defensive rejection of other information. Alternatively, it might represent a compartmentalized epistemic filter, whereby adolescents remain selectively open to certain sources—often driven by emotional needs or a sense of ingroup belonging—while mistrustful towards others. In either case, such ambivalence may position adolescents in a conflicted state, caught between social vulnerability and pervasive suspicion. Similar ambivalent profiles have been identified in adult clinical populations using person‐centered methods (Benzi et al. 2025), suggesting that maladaptive combinations of epistemic stances transcend diagnostic categories. Correspondingly, adolescents in Profile 1 reported the highest levels of internalizing problems, significantly surpassing all other groups. These findings imply that epistemic ambivalence—even in the absence of notable RF deficits—may increase vulnerability to chronic anxiety, rumination, and social threat sensitivity. This interpretation aligns with evidence that young people with internalizing difficulties often follow developmental trajectories of mentalising akin to their non‐clinical peers in early adolescence (Bizzi et al. 2019, 2021, 2025).

The Impaired RF profile (Profile 4), which accounts for about a quarter of the sample (24.9%), was characterized by substantial mentalising impairment. Adolescents in this group reported the highest scores on both Uncertainty and Certainty regarding mental states, alongside average scores on the ET indicators. The simultaneous presence of elevated Certainty and Uncertainty indicates a state of cognitive and emotional confusion, a pattern described in both theoretical models and clinical contexts (Martin‐Gagnon et al. 2023). The epistemic stances appeared to be in a state of static equilibrium, with no position dominating. This absence of epistemic anchoring could promote a random or indiscriminate approach to evaluating socially relevant information. Furthermore, this lack of a dominant epistemic position, combined with significant difficulties in mentalising, may reflect a diminished ability to integrate new knowledge and respond to social cues—particularly in distinguishing trustworthy from untrustworthy sources. Consistent with this interpretation, adolescents in this profile reported the highest levels of externalizing problems, supporting models that link RF deficits—especially an impaired capacity for reflecting on the impact of one's actions on others—to disruptive and aggressive behavior (Abate et al. 2017; Ensink et al. 2017; Knapen et al. 2025; Talia et al. 2021). This pattern of high RF impairment may represent a developmental phase where mentalising capacities are less developed, and the regulation of social–cognitive processes is less integrated—especially in the presence of increased externalizing tendencies.

The Adaptive profile (Profile 3), representing about a quarter of the sample (24.4%), closely matched the hypothesized adaptive group. It was characterized by above‐average Trust, the lowest levels of Mistrust, and low Credulity, with minimal impairment on both RF dimensions. This profile aligns with a developmental trajectory grounded in secure attachment, where flexible and resilient mentalising coexists with an open yet appropriately vigilant epistemic stance toward social learning (Allen 2018; Fonagy and Luyten 2009). Longitudinal studies support the idea that such profiles are linked to adaptive functioning throughout development (Desatnik et al. 2023). Similar patterns have also been observed in adult clinical samples, where adaptive ET combined with effective emotion regulation predicted more favorable functioning across transdiagnostic presentations (Benzi et al. 2025). The adaptive nature of this profile was confirmed by the MANOVA results, which indicated the lowest levels of both internalizing and externalizing problems.

Conversely, the Disengaged profile (Profile 2), the smallest group (18.5%), was characterized by uniformly low scores across all RF and ET indicators. Despite this generally flat profile, adolescents in this group reported low levels of psychopathology, similar to those in the Adaptive group. This configuration may reflect a disengaged social‐cognitive style, marked by reduced reflective involvement with mental states and limited openness to socially transmitted knowledge. Within an attachment framework, it may be understood as a distancing or deactivating orientation, in which social and emotional experience is minimized rather than actively processed. In this sense, the relative absence of symptoms should not be equated with fully adaptive or developmentally mature functioning. Rather, this profile may capture a mode of organization that remains apparently stable in a community context, but is less generative in terms of social learning, emotional elaboration, and psychological flexibility. Although not directly associated with psychopathology in the present sample, it may, therefore, reflect a more silent form of vulnerability, characterized less by overt distress than by reduced engagement with the interpersonal and affective processes that support development. This disengagement from interpersonal sharing, combined with emotional withdrawal and a failure to update self‐representations, might foster unexamined self‐reflection, creating a “blind spot” in self‐awareness. It is important to note that these interpretations are exploratory and should be validated through future longitudinal research.

The theoretical implications of these findings deserve further reflection. A striking finding of this study was the lack of a simple, linear relationship between RF and ET. This was not only reflected in the weak bivariate correlations but was also supported by our variable‐centered benchmark analyses, which examined several theoretically plausible mediation pathways (RF → ET → Psychopathology) and found no significant indirect effects. This empirical outcome is especially significant given the common assumption that mentalising is a necessary developmental precursor to ET (Fonagy and Allison 2014). Our findings imply that this relationship is more intricate than previously understood, particularly during adolescence, a period marked by neurocognitive reorganization and increased social sensitivity (Blakemore and Mills 2014). Recent socio‐epistemic reformulations of attachment theory are also relevant here. In this view, ET is not simply a downstream product of mentalising but a more fundamental developmental condition that influences how interpersonal communication is received, filtered, and generalized across different contexts. From this perspective, attachment‐related differences may be understood in terms of how trust in socially transmitted knowledge is established and managed, rather than assuming a straightforward developmental sequence in which mentalising necessarily comes before ET (Talia et al. 2026a, 2026b, 2026c). Although the present study does not directly test this theoretical model, our findings are broadly consistent with the view that epistemic stances may play a somewhat distinct role in organizing adolescents' socio‐cognitive functioning, rather than being entirely reducible to RF.

The data suggest that ET reflects a more stable, trait‐like stance shaped by early caregiving, while RF functions as a context‐dependent, state‐like process sensitive to arousal and situational demands (Fonagy and Allison 2023; Luyten et al. 2020). This distinction, previously observed in adult samples (Benzi et al. 2025), is increasingly supported by developmental evidence (Locati, Milesi, et al. 2023). From this perspective, middle childhood may serve as a preparatory phase in equipping individuals to handle the epistemic demands of adolescence (Locati et al. 2025). The failure of variable‐centered models to capture the associations between these constructs provides a strong empirical rationale for the person‐centered approach employed here. It was, therefore, not only a complementary but a necessary perspective, revealing that the relationship is not simply linear but rather that the specific patterning of RF and ET within individuals best explains psychological functioning. These findings call for a more integrated developmental model, viewing mentalising and ET as interdependent yet dissociable systems (Luyten et al. 2020). This interpretation is also consistent with recent work showing that mentalising and ET are embedded within broader systems of personality and self‐functioning, rather than operating as isolated mechanisms (Benzi et al. 2026).

Identifying these profiles might have important clinical implications, challenging a “one‐size‐fits‐all” approach and emphasizing the need for person‐centered assessment and treatment. Although these findings are preliminary, they suggest that profile‐based differences in RF and ET may have relevance for both prevention and intervention. More broadly, this interpretation is consistent with recent relational and generative models of psychopathology, which conceptualize maladaptive functioning as emerging from different patterns of self–other inference and social learning (Zavlis et al. 2026). At present, such implications should be considered hypothesis‐generating rather than definitive and will require replication and longitudinal validation before they can meaningfully inform clinical decision‐making. For example, an adolescent's profile of social‐cognitive strengths and weaknesses may help identify different intervention priorities. An adolescent from the Impaired Reflective Functioning (Profile 4) group, whose difficulties mainly stem from severe RF impairment, may particularly benefit from interventions targeting basic mentalising skills and affect regulation, such as Mentalisation‐Based Treatment for Adolescents (MBT‐A) (Rossouw et al. 2021; Sharp and Rossouw 2024). Conversely, an adolescent from the Ambivalent Epistemic Stances (Profile 1) group, who exhibits relatively intact RF but marked epistemic conflict, may benefit more from interventions aimed at strengthening the capacity to identify and rely on reliable, personally relevant information. For these adolescents, working on the integration of coexisting tendencies toward Credulity and Mistrust may help promote a more balanced epistemic stance. Interventions for this group may usefully address interpersonal and identity‐related patterns associated with these conflicting tendencies, with the aim of reducing maladaptive epistemic stances linked to internalizing distress (Li et al. 2023; Nolte et al. 2023). Furthermore, although adolescents in the Disengaged (Profile 2) group may not report significant distress, their profile suggests a possible vulnerability that could be relevant for prevention. Their relatively immature social‐cognitive functioning may benefit from interventions that address mistrust and support greater openness to new knowledge and reflective engagement. In this sense, preventive work might focus on gradually fostering interpersonal trust and validating emotional and mental states, thereby supporting the development of mentalisation and a more coherent sense of self.

### 4.1 Limitations and Future Directions

The limitations of this study should be recognized. First, the cross‐sectional design prevents conclusions about causality or developmental trajectories. Therefore, interpretations involving developmental processes are tentative and need confirmation through longitudinal studies. Second, the study omits significant contextual variables such as socioeconomic status, family environment, trauma history, and current life stressors, all of which are highly relevant to theories of RF and ET. Their absence limits the interpretation of some profiles, especially the Disengaged group. Indeed, an alternative interpretation is that adolescents in this profile may not have been experiencing particularly high interpersonal or emotional demands at the time of assessment. In this case, the combination of low symptoms and low engagement with social‐cognitive processes might reflect contextual conditions rather than genuinely adaptive functioning. Since the present study did not assess contextual stressors or current life challenges, this possibility cannot be ruled out. Third, our dependence on self‐report measures is vulnerable to reporting biases, and the internal consistency for the ETMCQ Trust and Mistrust subscales was moderate, although consistent with other studies (Campbell et al. 2021; Liotti et al. 2023). More broadly, both the RFQY‐13 and ETMCQ lack well‐established and reliable cut‐offs. Accordingly, the profiles identified in the present study should be interpreted as relative, sample‐specific configurations rather than as normative or clinically validated categories. Lastly, since this was a community sample, replication in clinical populations is necessary to determine whether these profiles and their associated risks differ among youth with formal diagnoses. Future research should build on these findings by using longitudinal designs to observe how these profiles develop and change over time, and incorporate multi‐method assessments to supplement self‐report data. An especially promising approach would be to reverse the analytical process: first categorizing adolescents by symptom profiles (e.g., internalizing, externalizing, comorbid, low symptom) and then testing whether RF and ET configurations consistently differentiate these groups. This would enable an evaluation of how well these social‐cognitive configurations correspond to established categories of psychopathology.

## 5 Conclusion

This study is the first, to our knowledge, to apply a person‐centered approach to model the complex interplay between RF and the triadic components of ET in adolescents, as well as to investigate how these relate to adolescent psychopathology. Our findings go beyond a variable‐centered perspective, unveiling four distinct social‐cognitive profiles, each with a unique connection to mental health. The results highlight that adolescent psychopathology cannot be understood through a single nosographic framework; instead, it appears as a heterogeneous and multidimensional phenomenon. A detailed understanding of an individual's specific profile of mentalising abilities and epistemic stances is essential for further advancing developmental theory and developing more targeted and effective clinical interventions.

## Author Contributions

**Ilaria Maria Antonietta Benzi:** conceptualization, writing – original draft, methodology, writing – review and editing, formal analysis. **Nick Midgley:** writing – review and editing. **Karin Ensink:** writing – review and editing. **Laura Parolin:** writing – review and editing. **Peter Fonagy:** writing – review and editing. **Francesca Locati:** conceptualization, writing – original draft, writing – review and editing, data curation, supervision, investigation.

## Funding

The authors have nothing to report.

## Ethics Statement

The study was approved by the Ethics Committee of the University of Milano‐Bicocca and was conducted in accordance with the Declaration of Helsinki. Informed consent was obtained from all individual participants and their parents or legal guardians.

## Conflicts of Interest

The authors declare no conflicts of interest.

## Data Availability

De‐identified data and analytic code are available from the corresponding author upon reasonable request.
